# Identification and Characterization of Cuticular Proteins in the Miridae Insect *Apolygus lucorum*

**DOI:** 10.3390/ijms27073178

**Published:** 2026-03-31

**Authors:** Hui Zhang, Kaicheng Han, Han Gao, Ying Tian, Chengjun Li, Bin Li

**Affiliations:** Jiangsu Key Laboratory for Biodiversity and Biotechnology, College of Life Sciences, Nanjing Normal University, Nanjing 210023, China

**Keywords:** cuticular proteins, expression patterns, insecticide resistance

## Abstract

Insect cuticles exhibit remarkable resistance to environmental stresses, largely attributed to cuticular proteins (CPs), which are crucial for insect development and adaptation. CPs also contribute to insecticide resistance, making them a key focus in molecular entomology. *Apolygus lucorum*, a globally distributed omnivorous pest within the Miridae family (Hemiptera: Heteroptera), inflicts significant economic losses by damaging a wide range of crops. However, information on CPs in the Miridae family remains scarce, limiting our understanding of their molecular mechanisms of adaptation and resistance. Here, we performed a genome-wide identification of CPs in *A. lucorum* and reanalyzed transcriptomic data under insecticide exposure to identify resistance-related candidates. A total of 211 CPs were identified and classified into 10 subfamilies. Notably, the RR-2, Tweedle, and CPF families showed significant expansions compared to other hemipterans, likely driven by tandem duplication events, which may contribute to *A. lucorum*’s broad environmental adaptability and host range. Expression profiling revealed two major patterns: one with peak expression during the nymphal stage and another maintained throughout the entire life cycle. Crucially, 75 CPs were upregulated following insecticide treatment, underscoring their potential role in resistance and their value as targets for pest control. Our findings provide a comprehensive foundation for future studies on the molecular functions of CPs in *A. lucorum* and their involvement in insecticide resistance, paving the way for novel management strategies.

## 1. Introduction

The insect cuticle, an intricate extracellular structure secreted by epidermal cells, functions as the primary outer barrier of the body. It plays essential roles in maintaining morphology, enabling movement, and supporting growth, while also serving as the first line of defense against environmental stresses such as desiccation, mechanical damage, pathogens, and insecticides [1]. The cuticle can protect the insect against insecticides; previous research has reported that insect cuticle can prevent or delay the penetration of insecticides into the body by thickening the cuticle or altering their cuticular structure and composition, and this is one of the mechanisms for insecticide resistance [2,3,4]. The insect cuticle consists of the envelope, epicuticle and procuticle, which is made up of the exocuticle and endocuticle and is primarily composed of chitin and several types of cuticular proteins (CPs).

The first insect genome assembly was that of *Drosophila melanogaster* [5], with the rapid improvement of next-generation sequencing technology, a large number of genomes have become available and the CPs were identified in *Plutella xylostella* [6], *Manduca sexta* [7], *Spodoptera litura* [8], *Pteromalus puparum* [9], *Bombyx mori* [10], *Dendrolimus punctatus* [11], *Anopheles sinensis* [12], *Bactrocera dorsalis* [13]. These investigations have shown that there is a significant difference in quantity and categories of CPs across various insect species. According to earlier studies, the number of genes that code for CPs typically makes up more than 1% of all the protein-coding genes in an insect genome [14]. Based on conserved motifs, CPs can be categorized into 13 families, including CPR, CPAP, Tweedle, CPLCA, CPLCG, CPLCW, CPLCP, CPF, CPFL, CPCFC, 18aa, CPG, and Apidermin [15,16]. Most insect CPs feature the Rebers and Riddiford (R&R) chitin-binding domain. The CPRs can be further classified into three sub-forms: RR-1, RR-2, and RR-3 [10,17,18]. The flexible cuticles of the larval stages included RR-1 proteins in great abundance, while the rigid cuticles of the pupal or adult stages had RR-2 proteins in great abundance [6,19]. CPAP proteins also contain chitin-binding domains and are further categorized into CPAP1 and CPAP3 subfamilies based on the number of peritrophin A-type (ChtBD2) domains [20,21]. The Tweedle family comprises proteins with four conserved blocks of approximately 100 amino acids each, first identified in *D. melanogaster* through a body-shape mutant phenotype [22]. According to the unique sequence properties, the cuticular protein of low complexity families (CPLC) are divided into CPLCA, CPLCG, CPLCW, and CPLCP, which are each abundant in AAP (A/V) repeats, glycine, tryptophan, and P (V/Y) residues, respectively [16]. The CPF family is defined by a conserved 44 amino acid motif, while CPF-like (CPFL) lacks this domain but retains a conserved C-terminal sequence [23,24]. The CPCFC, 18aa, CPG families contain two or three repeats of the C-x(5)-C motifs, an 18aa motif, glycine repeats, respectively [6]. The Apidermin (APD) family was recorded in *Apis mellifera*, which consists of three extremely hydrophobic proteins containing at least 30% alanine [16].

*Apolygus lucorum* (Meyer-Dür) (Hemiptera: Miridae) is a globally agricultural pest and notorious for causing severe damage to a wide range of crops and substantial economic losses [25]. Current management of *A. lucorum* mainly relies on chemical pesticides, which frequently result in major environmental and safety issues. Therefore, developing other environmentally friendly approaches is considered a top priority for managing this pest [8]. Understanding the molecular processes of insecticide resistance is fundamental to overcoming this difficulty. Notably, cuticular modification—such as thickening or compositional change—has been linked to reduced cuticular penetration of insecticides, a resistance mechanism largely attributed to the upregulation of multiple cuticular protein (CP) genes [2,6]. Recent research in *P. xylostella* showed that 18 CP genes were up-regulated more than two-fold in chlorpyrifos-resistant strains, and several CP genes were induced following chlorpyrifos treatment [6]. However, the understanding of *A. lucorum* CPs is limited, and whether it can affect the development of insecticide resistance is still unclear. Systematic identification and expression profiling of CPs thus represent an essential first step toward elucidating their functional significance in the development and resistance of *A. lucorum.*

In this study, we performed a genome-wide identification and characterization of CP families in *A. lucorum* using the published genome sequence [26]. Subsequently, phylogenetic analysis of CPs between *A. lucorum* and other hemipteran insects (*Nilaparvata lugens* and *Acyrthosiphon pisum*) was conducted to study the evolutionary patterns of CPs. Additionally, the expression pattern of CP genes at various developmental stages and CP genes linked to insecticide resistance were examined using public transcriptome data [26]. The findings of this study lay the groundwork for further investigations into the CP function of *A. lucorum*.

## 2. Results

### 2.1. Identification and Classification of CP Genes

**Figure 1:**
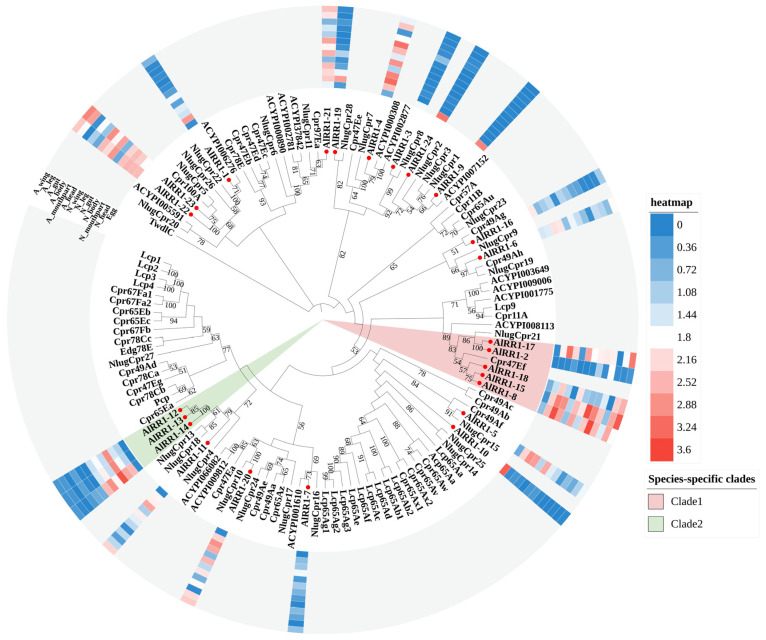
Phylogenetic tree reconstruction of RR-1 type CPR gene family from *D. melanogaster*, *N. lugens*, *A. pisum* and *A. lucorum* (red circle) inferred from maximum likelihood (ML). The numbers on the tree were the bootstrap values (values below 50 are not shown). The tree was rooted by the *D. melanogaster TwdlC*. The expression profile of *A. lucorum* RR-1 genes from different tissues is displayed on the right side of the phylogenetic tree. The transcription level of each gene is represented by a square with a color that codes for the values of lg (TPM + 1). Red indicates high expression, whereas blue represents low expression. N, nymph; A, adult.

**Figure 2:**
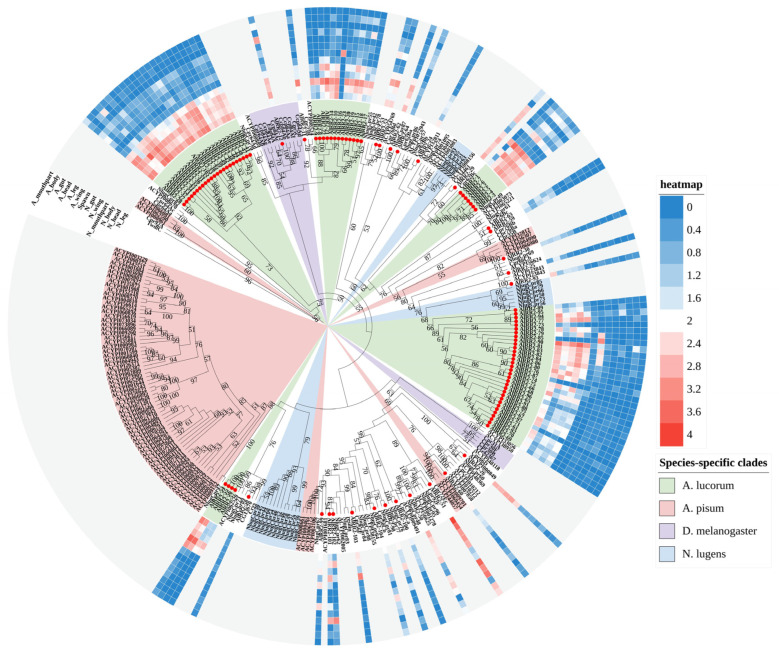
Phylogenetic tree reconstruction of RR-2 type CPR gene family from *D. melanogaster*, *N. lugens*, *A. pisum* and *A. lucorum* (red circle) inferred from maximum likelihood (ML). The numbers on the tree were the bootstrap values (values below 50 are not shown). The tree was rooted by the *D. melanogaster TwdlC*. The expression profile of *A. lucorum* RR-2 genes from different tissues is displayed on the right side of the phylogenetic tree. The transcription level of each gene is represented by a square with a color that codes for the values of lg (TPM + 1). Red indicates high expression, whereas blue represents low expression. N, nymph; A, adult.

**Figure 3:**
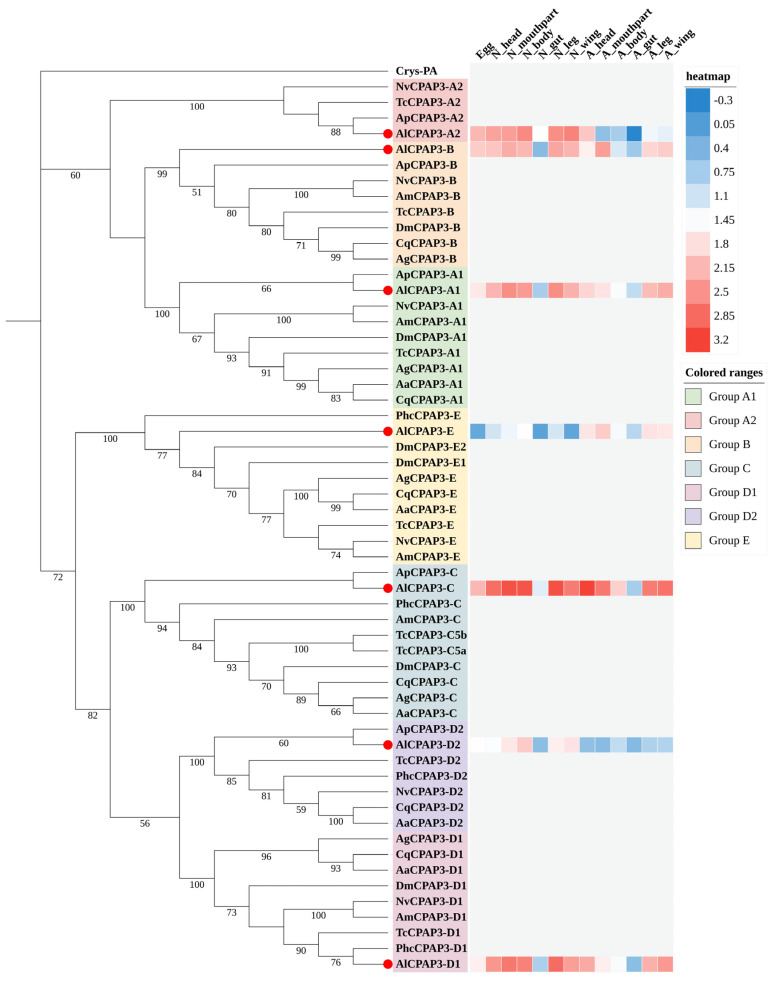
Phylogenetic analysis of CPAP3s from *A. lucorum* and nine other different insect species: *Acyrthosiphon pisum* (Ap), *Aedes aegypti* (Aa), *Anopheles gambiae* (Ag), *Apis mellifera* (Am), *Culex quinquefasciatus* (Cq), *Drosophila melanogaster* (Dm), *Nasonia vitripennis* (Nv), *Pediculus humanus corporis* (Phc), *Tribolium castaneum* (Tc). CPAP3 genes from *A. lucorum* are indicated with red circle. The numbers on the tree were the bootstrap values (values below 50 are not shown). The tree was rooted by the *D. melanogaster Crys-PA*. The expression profile of *A. lucorum* CPAP3 genes from different tissues is displayed on the right side of the phylogenetic tree. The transcription level of each gene is represented by a square with a color that codes for the values of lg (TPM + 1). Red indicates high expression, whereas blue represents low expression. N, nymph; A, adult.

**Figure 4:**
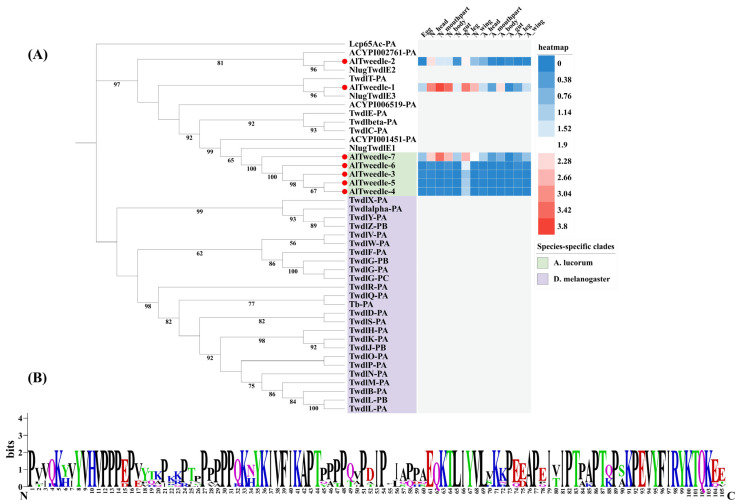
(**A**) Phylogenetic tree reconstruction of Tweedle gene (TWDL genes) family from *D. melanogaster*, *N. lugens*, *A. pisum* and *A. lucorum* (red circle) inferred from maximum likelihood (ML). The numbers on the tree were the bootstrap values (values below 50 are not shown). The tree was rooted by the *D. melanogaster Lcp65Ac-PA*. The expression profile of *A. lucorum* Tweedle genes from different tissues is displayed on the right side of the phylogenetic tree. The transcription level of each gene is represented by a square with a color that codes for the values of lg (TPM + 1). Red indicates high expression, whereas blue represents low expression. N, nymph; A, adult. (**B**) The sequence logo shows the conserved region of the Tweedle proteins from *A. lucorum*.

**Figure 5:**
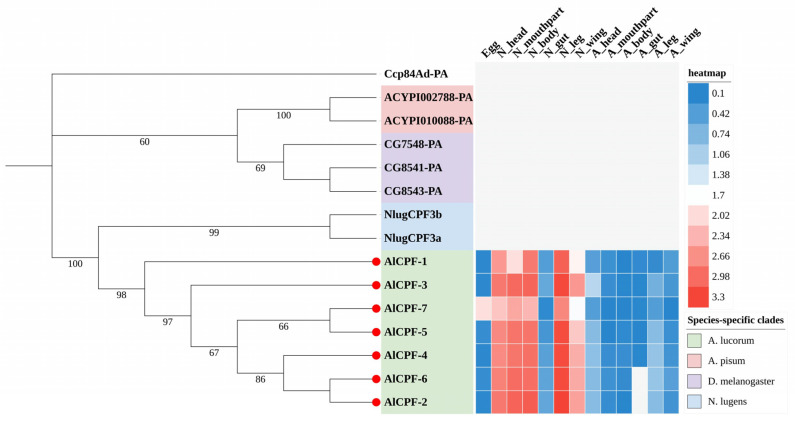
Phylogenetic tree reconstruction of CPF gene family from *D. melanogaster*, *N. lugens*, *A. pisum* and *A. lucorum* (red circle) inferred from maximum likelihood (ML). The numbers on the tree were the bootstrap values (values below 50 are not shown). The tree was rooted by the *D. melanogaster Ccp84Ad-PA*. The expression profile of *A. lucorum* CPF genes from different tissues is displayed on the right side of the phylogenetic tree. The transcription level of each gene is represented by a square with a color that codes for the values of lg (TPM + 1). Red indicates high expression, whereas blue represents low expression. N, nymph; A, adult.

**Figure 6:**
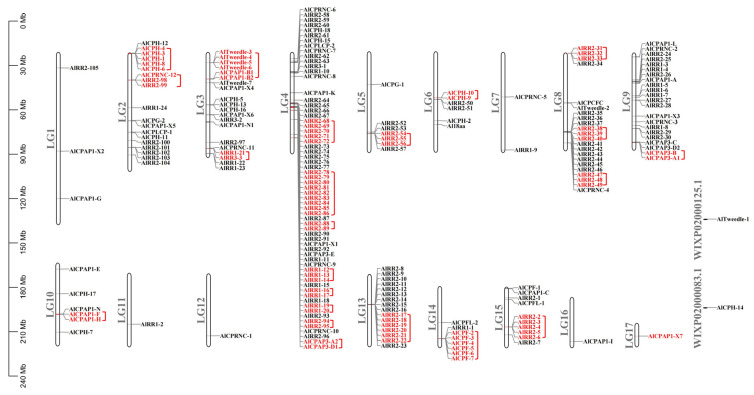
Chromosomal locations and tandem duplicated gene pairs of the 211 putative cuticular protein genes. Each was mapped to the chromosome based on its physical location. The chromosome number is indicated at the left. The tandem duplicated genes were outlined in red.

**Figure 7:**
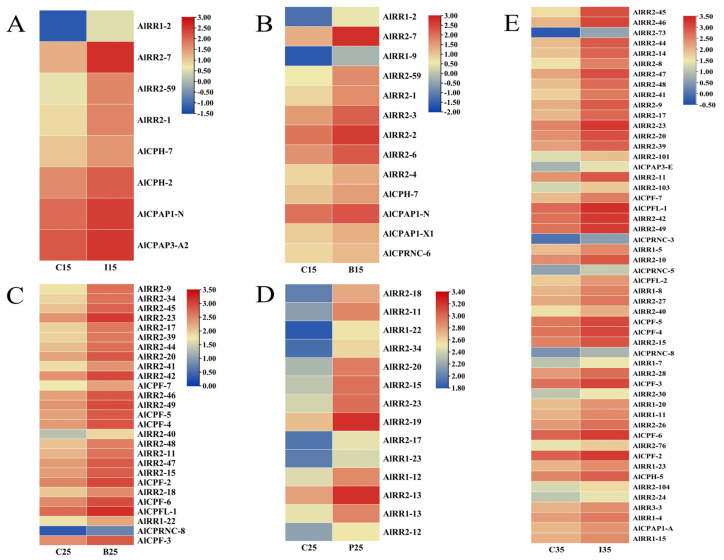
The insecticide stress expression profile of up-regulated cuticular protein genes after insecticide exposure in *A. lucorum*. Expression levels of different temperatures and insecticide treatments are represented as a heatmap. Red indicates high expression and blue indicates low expression. C, control; I, imidachloprid; B, beta-cypermethrin; P, phoxim. (**A**) C15 vs. I15, (**B**) C15 vs. B15, (**C**) C25 vs. B25, (**D**) C25 vs. P25, (**E**) C35 vs. I35.

A total of 211 putative CP sequences were identified and classified in *A. lucorum* (Table 1). These CPs belonged to ten different groups: CPR, CPAP1, CPAP3, Tweedle, CPF, CPFL, CPCFC, CPG, CPLCP, and 18 aa. Additionally, based on the sequence similarity and signal peptide with the silkworm CPH proteins [10], a set of unclassified CPs were classified as CPH. Detailed information about CP genes in *A. lucorum* is presented in Table S1. Phylogenetic analysis of CPs between *A. lucorum* and other insects was conducted to study the evolutionary patterns of CPs. The expression profiles of each CP gene across various tissues and developmental stages were determined using transcriptome data (Figure 1, Figure 2, Figure 3, Figure 4 and Figure 5). The chromosomal positions of all CPs are depicted in Figure 6. Furthermore, the expression patterns of potential resistance-associated CPs obtained from transcriptome data are shown in Figure 7.

### 2.2. The CPR Gene Family

The CPR (Cuticular Proteins with R&R consensus) family is widely recognized as the most prevalent and abundant CP family among insects [16]. The number of CPR genes varies significantly across different insect lineages, with 96 in *N. lugens* (*Hemiptera: Delphacidae*) [14], 125 in *A. pisum* (*Hemiptera: Aphididae*) [27], and 101 in *D. melanogaster* (Diptera: Drosophilidae) [28]. In our study, we identified 144 CPR genes, constituting 68% of the CP genes in *A. lucorum* (Table 1). Among them, 24 were assigned to RR-1 subfamily and 105 to RR-2 subfamily using CuticleDB. We manually annotated three RR-3 subfamily proteins based on their similarity to known RR-3 proteins in *B. mori* [10]. The remaining 12 CPR sequences, which couldn’t be categorized into any subfamily, were designated as RRNC (Table S1). Subsequently, phylogenetic trees for the RR-1 and RR-2 subfamilies were reconstructed (Figure S1). As anticipated, the RR-1 and RR-2 subfamilies formed distinct clades, consistent with previous reports [14,28] in *N. lugens* and *D. melanogaster*. However, notably, according to CuticleDB, several CPR genes should belong to the RR-2 subfamily, but they fell into the RR-1 cluster (Figure S1).

#### 2.2.1. RR-1 Subfamily

As the RR-1 and RR-2 subfamily proteins of *A. lucorum* formed paraphyletic clades, we established phylogenetic trees for these two types of proteins separately (Figure 1 and Figure 2) by using the extended R&R domains from *A. lucorum*, *D. melanogaster*, *N. lugens*, and *A. pisum*. In combination with the phylogenetic tree, we identified 9 RR-1 proteins that exhibited one-to-one orthologous relationships with other species. Among these, 7 were orthologous to hemipteran species (*A. pisum*, *N. lugens*), and 2 were orthologous to non-hemipteran species (*D. melanogaster*). The remaining RR-1 proteins formed two distinct evolutionary clades on the tree (Clade1-2). Notable examples include *AlRR1-2, AlRR1-8, AlRR1-15*, *AlRR1-17*, *AlRR1-18* and *AlRR1-11*, *AlRR1-12*, *AlRR1-13*, *AlRR1-14*, which each formed species-specific clades (Figure 1). Within the RR-1 subfamily, 12 members displayed substantial expression in more than half of the tissues (*AlRR1-4*, *AlRR1-5*, *AlRR1-6*, *AlRR1-8*, *AlRR1-11*, *AlRR1-15*, *AlRR1-17*, *AlRR1-18*, *AlRR1-20-23*), with 7 members (*AlRR1-4*, *AlRR1-5*, *AlRR1-11-15*) exhibiting high expression during the nymph stage. Additionally, *AlRR1-4*, *AlRR1-8*, *AlRR1-15*, *AlRR1-17* displayed elevated expression in all tissues except for eggs and guts. *AlRR1-9*, *AlRR1-10*, and *AlRR1-24* were significantly expressed only in eggs, apart from other tissues.

#### 2.2.2. RR-2 Subfamily

The RR-2 proteins represent the largest group within the CPR family and are the most numerous across various insects. Unlike the RR-1 subfamily, RR-2 exhibits remarkable sequence and length conservation. Phylogenetic analysis revealed that only a limited number of RR-2 members displayed one-to-one orthologous relationships with other species, including seven with *N. lugens* and six with *D. melanogaster* clades, a pattern similarly observed in three other species examined. In contrast, most remaining RR-2 proteins cluster into distinct species-specific clades. Specifically, within the RR-2 subfamily, we identified five *A. lucorum*-specific, five *A. pisum*-specific, three *N. lugens*-specific, and two *D. melanogaster*-specific clades (Figure 2). Expression profiling revealed that only 24 RR-2 genes are expressed in over half of the examined tissues, with the majority exhibiting peak expression during the nymph stage. Notably, *AlRR2-58* and *AlRR2-87* showed low expression in all tissues; *AlRR2-16* and *AlRR2-85* exhibited significant expression exclusively in eggs. Additionally, all RR-2 members showed low expression in the adult guts.

### 2.3. The CPAP Gene Family

The CPAP family is the second-largest CP subfamily, following the CPR family, and is distinguished by a motif featuring six distinctly spaced cysteine residues known as the type 2 chitin-binding domain (ChtBD2) [29,30]. CPAPs are categorized based on the number of peritrophin type A chitin-binding domains they possess, and they fall into two subgroups: CPAP1 and CPAP3, characterized by having one and three ChtBD2s, respectively. Within the *A. lucorum* genome, we identified a total of 27 potential CPAPs, consisting of 20 CPAP1 proteins and seven CPAP3 proteins.

#### 2.3.1. CPAP1 Subfamily

In this study, 20 CPAP1 proteins were identified in *A. lucorum*, namely *AlCPAP1-A*, *AlCPAP1-B1*, *AlCPAP1-B2*, *AlCPAP1-C*, *AlCPAP1-E*, *AlCPAP1-F*, *AlCPAP1-G*, *AlCPAP1-H*, *AlCPAP1-I*, *AlCPAP1-K*, *AlCPAP1-L*, *AlCPAP1-N*, *AlCPAP1-N1* and *AlCPAP1-X1-7*. Phylogenetic analysis revealed that CPAP1 proteins were clearly clustered into distinct groups on the tree that includes representative members from various insect orders (Figure S2). Furthermore, our phylogenetic analysis indicated that 12 CPAP1 proteins in *A. lucorum* were conserved in insects, while CPAP1-D, CPAP1-J, CPAP1-M, and CPAP1-O proteins were notably absent. Among them, CPAP1-D was exclusively found in *Tribolium castaneum*. Additionally, in contrast to other insects, CPAP1-N contained two paralogs (*AlCPAP1-N*, *N1*) in *A. lucorum*. Moreover, there were seven proteins named CPAP1-X that exhibited high homology with known CPAP1 proteins but couldn’t be confidently clustered into any group. Expression profile analysis revealed that seven members exhibited high expression levels in more than half of the tissues. *AlCPAP1-B2* and *AlCPAP1-X5* displayed low expression across all tissues, while *AlCPAP1-X7* exhibited the highest expression in the nymph guts.

#### 2.3.2. CPAP3 Subfamily

Barry et al. (1999) first identified a gene encoding a peritrophin-like protein in the embryonic tracheae of *D. melanogaster* and named it “*gasp*” [31]. Subsequently, more proteins with similar characteristics were identified in *D. melanogaster*, collectively known as the “*obstructor*” multigene family [32]. The *gasp*/*obstructor* genes for *T. castaneum* were designated as “*CPAP3*”. The CPAP3 gene family can be further subdivided into two groups, CPAP3-A-E and CPAP3-F-J. CPAP3-A-E have been identified in various insects, while CPAP3-F-J have been exclusively described in *D. melanogaster* [29,32]. When compared to the CPAP1 genes, the CPAP3 genes exhibit a higher degree of conservation and orthologs to the seven described genes (CPAP3-A1, CPAP3-A2, CPAP3-B, CPAP3-C, CPAP3-D1, CPAP3-D2, and E) can be found in different insect species such as *C. capitata*, *T. castaneum*, *B. mori*, *M. sexta*, *A. pisum* and *A. mellifera* [13]. Consistent with other insects, seven conserved CPAP3 proteins of *A. lucorum* were annotated successfully in the genome database, comprising *AlCPAP3-A1*, *AlCPAP3-A2*, *AlCPAP3-B*, *AlCPAP3-C*, *AlCPAP3-D1*, *AlCPAP3-D2*, *AlCPAP3-E*. Notably, CPAP3-E in *D. melanogaster* contained two paralogs (CPAP3-E1, E2), whereas only *AlCPAP3-E* was identified in *A. lucorum*. It’s worth mentioning that CPAP3-C has two alternatively spliced forms in *T. castaneum* (*TcCPAP3-C5a*, *TcCPAP3-C5b*). Additionally, CPAP3 proteins in *A. lucorum* consistently cluster with those from *A. pisum*, forming one-to-one orthologous relationships (Figure 3). Expression profile analysis showed that all CPAP3 members exhibited high expression levels in over half of the tissues. *AlCPAP3-C* displayed significant expression in all tissues except for the nymph guts, with the highest expression in the adult heads. Interestingly, all members exhibited low expression in the adult guts.

### 2.4. The Tweedle Gene Family

The Tweedle gene family was originally identified in *D. melanogaster* through mutations affecting larval body shape, notably TweedleD, which results from inadequate cuticle thickness and impairs the maintenance of normal larval morphology [13,22]. Here, we identified seven Tweedle genes in *A. lucorum.* This number exceeds that found in other hemipteran species, with *N. lugens* and *A. pisum* each possessing three genes encoding Tweedle proteins (Table 1). Phylogenetic analysis revealed that five of the *A. lucorum* Tweedle genes clustered into a species-specific clade, while the remaining two showed one-to-one orthology with *N. lugens*. Although the number of Tweedle members differs markedly between *D. melanogaster* and *A. lucorum*, all *Drosophila* Tweedle genes formed two Diptera-specific clades in our phylogeny (Figure 4A), consistent with observations by Zhou et al. [12], and indicative of independent evolutionary trajectories across insect orders. Broadly, the phylogenetic tree is separated into two major branches corresponding to *Hemipteran* and *Dipteran* lineages, suggesting that Tweedle genes experience independent evolutionary pressures across insect taxa. Sequence analysis confirmed that Tweedle proteins contain four conserved structural blocks [22]. WEBLOGO alignment of the seven *A. lucorum* Tweedle sequences clearly delineated these blocks (Figure 4B): Block I features a KX2Y/F motif (with X2 representing two non-conserved amino acids), block II displays a KX4-5FIKAP sequence, block III showcases a TX2YVL motif, and block IV consists of a KPEVYXFV/IKY sequence. These conserved regions are structurally important, as prior work has shown that the β-strand-rich half-barrel architecture of Tweedle proteins allows aromatic residues to interact with chitin [33]. Expression profile analysis indicates that *A. lucorum* Tweedle genes generally exhibit low expression levels during the adult stage. Meanwhile, *AlTweedle-1* and *AlTweedle-7* exhibit high expression throughout the entire nymph stage, with *AlTweedle-1* reaching its peak expression in the nymph mouthparts. Additionally, the expression levels of Tweedle genes in the nymphal legs are generally higher than those in other tissues.

### 2.5. The CPF Gene Family

The CPF proteins were initially characterized by a conserved 51 amino acid motif that was identified from six CPs of *Tenebrio molitor* and *L. migratoria* [34]. However, this original consensus has undergone significant modifications, reducing the conserved region to 42–44 amino acids through the alignment of an expanded dataset of CPF proteins from various insect species [24]. In our investigation, we also identified seven members of the CPF family proteins in *A. lucorum*. Phylogenetic analysis unveiled that CPF genes from *A. lucorum*, *N. lugens*, *A. pisum*, and *D. melanogaster* formed distinct branches, as shown in Figure 5. Combined with expression profile analysis, it becomes evident that all CPF genes exhibit low expression during the adult stage, while displaying high expression in nymph tissues, excluding the guts. Remarkably, only one gene, *AlCPF-7*, exhibits high expression levels in eggs.

### 2.6. Chromosome Locations and Duplication Events

By MCScanX analysis, we perceived 51 tandem duplication events occurred among CPR, CPAP, Tweedle, CPF and CPH gene families (Figure 6). Notably, these duplication events were predominantly concentrated on LG (linkage group) 2, 3, 4, 5, 6, 8, 9, 10, 13, 14, and 15. Among these, LG4 hosted the highest number of genes involved in duplication events. Remarkably, the majority of CPR genes were situated on linkage group 4 (LG4), with 25 CPR genes forming 8 tandem arrays. Furthermore, up to 43 RR-2 members underwent tandem duplication. Additionally, 25 RR-2 members were organized into tandem arrays comprising 2 to 6 genes on five linkage groups (LG2, LG5, LG8, LG13, LG15). An intriguing observation is the absence of co-existing RR-1 and RR-2 genes within the same tandem array, consistent with findings reported for *A. gambiae* by Cornman et al. [35]. Within the CPAP1 subfamily, two tandem duplication events were detected involving *AlCPAP1-B1* and *AlCPAP1-B2*, as well as *AlCPAP1-F* and *AlCPAP1-H*. In contrast, only one tandem duplication event was found in the CPAP3 subfamily, involving *AlCPAP3-A1* and *AlCPAP3-B*. Furthermore, *AlTweedle-3-AlTweedle-6* formed a tandem array on LG3, while *AlCPF-2-AlCPF-7* clustered in a tandem array on LG14.

### 2.7. Identifying CPs Associated with Insecticide Resistance

We conducted a thorough analysis by matching the identified CP genes against *A. lucorum* transcriptome data from various treatment groups (IM15, IM25, IM35, BCP15, BCP25, BCP35, PHX15, PHX25, and PHX35) to pinpoint differentially expressed genes (DEGs) [36]. Following RNA-seq analysis of these 9 groups of data, we successfully singled out cuticular protein genes from the pool of differentially expressed genes. With the same threshold screening criteria, it’s noteworthy that no CPs exhibited a response in the treatments involving beta cypermethrin and phoxim at 35 C. Additionally, two out of the remaining seven groups had no up-regulated CPs. Therefore, we concentrated on analyzing the up-regulated CPs in these five treatment groups, which are further detailed in Table S2. In total, we identified 75 significantly differentially expressed CP transcripts within these five treatments (IM15, BCP15, BCP25, PHX25, IM35) (Figure 7). A majority of these up-regulated genes belong to the CPR, CPF, and CPAP families. Notably, the expression profile demonstrated that some RR-2 genes were concurrently associated with resistance to multiple insecticides. Additionally, *AlCPAP3-E* exhibited up-regulation at 35 °C when treated with imidacloprid, and it had previously been reported as up-regulated in a deltamethrin-resistant strain of *A. sinensis* [12]. However, the potential role of CPAP3 subfamily members in insect resistance remains unconfirmed, and the role of CPAP3-E in insecticide resistance necessitates further investigation.

## 3. Discussion

In this study, a total of 211 putative CPs was identified in *A. lucorum* based on sequence characterisation or homology with other known CPs. All identified CP genes belong to 10 different families: CPR, CPAP1, CPAP3, Tweedle, CPF, CPFL, CPLCP, CPG, CPCFC and 18aa. These findings highlight the presence of several CP groups that are widespread across arthropods, while others exhibit restrictions to specific insect lineages. For example, the Apidermin family seems to be exclusive to Hymenoptera, whereas CPLCW family members are only found in Diptera, specifically within the family Culicidae (mosquitoes). Furthermore, the number of CP genes varies significantly among different species, ranging from 69 *A. mellifera* to 345 in *Aedes aegypti* [13]. These observations underscore the complexity of the insect CP superfamily. The differences in CP gene abundance among species may be attributed to their distinct ecological niches, as previously suggested [16]. The small number of CP genes in Hymenoptera (*A. mellifera* and *Nasonia vitripennis*) was due to their larvae living in protected environment [16]. In contrast, Lepidopteran insects possess a greater number of CP genes, which may be associated with the need to develop diverse cuticular tissues for protection in complex environments. *Apolygus lucorum*, a significant agricultural pest, is characterized by its strong environmental adaptability, rapid growth, and high dispersal capability. Among its noteworthy features, its rapid dispersal plays a crucial role in occupying a prominent ecological niche in cotton fields.

*Apolygus lucorum* has a diverse host range of over 200 plant species, including numerous important crops and fruits. This wide array of host plants enhances its environmental adaptability. Additionally, both nymphs and adults of *A. lucorum* can feed on crops throughout their life stages, relying on their highly developed olfactory systems for host switching. Consequently, *A. lucorum* inhabits a complex environment, necessitating the development of various cuticular tissues for self-protection. Thus, the expansion of CPs in *A. lucorum* might be linked to its environmental adaptability and the broad range of hosts it exploits.

Interestingly, a total of 144 CPRs were identified in *A. lucorum*. Meanwhile, previous phylogenetic studies have reported that CPR genes—particularly those in the RR-2 subfamily—often form species-specific clades. Examples include three RR-1 and seven RR-2 clusters in *B. mori* [10], six RR-2 groups in *D. punctatus* [11], and five RR-2 clades in *B. dorsalis* [13]. Consistently, our maximum likelihood trees showed that both RR-1 (Figure 1) and RR-2 (Figure 2) proteins in *A. lucorum* also assembled into multiple species-specific clades. This pattern was not unique to *A. lucorum*; similar within-species clustering, especially within the RR-2 subfamily, was observed in *N. lugens*, *A. pisum*, and *D. melanogaster*. The sequences within each species-specific clade were highly similar, likely originating from gene duplication events followed by independent expansion after speciation [7,13]. Studies in *Anopheles* and *Drosophila* have further shown that genes in such clusters often share high sequence identity and evolve in a concerted manner [28,35,37]. To investigate the genomic basis of clustering in *A. lucorum*, we performed synteny analysis using MCScanX. This revealed that tandem duplication represents the predominant mode of CP gene expansion in this species. The resulting gene clusters were distributed as tandem arrays across five chromosomes, with the largest array located on LG4 (Figure 6). Tandem duplication is a well-established mechanism for gene family expansion across diverse taxa [38]. In line with the pattern observed for RR-2, species-specific clades were also prominent in the Tweedle and CPF subfamilies, with clusters arranged in tandem arrays on LG3 and LG14. These results indicate that species-specific gene clusters significantly contribute to the overall CP repertoire in *A. lucorum* and that CP expansion frequently occurs via clustered duplication events. Notably, however, not all highly similar CP genes are physically adjacent within the same tandem array, suggesting that mechanisms beyond simple tandem duplication or deletion also contribute to the generation of sequence similarity among CPs.

Duplicated genes typically encounter diverse fates, including pseudogenization, subfunctionalization, or neofunctionalization. In contrast, tandemly duplicated genes tend to retain similar functions to their ancestral copies due to the sharing of regulatory elements, as evidenced in various instances [38]. The expression profiles of CP genes from the RR-2, Tweedle, and CPF families predominantly indicate their activity during nymph stages. In light of previous research, it can be speculated that these duplicated genes may play pivotal roles during the nymph stages. For example, when *dsNlugCpr47* (RR-2) was injected, approximately 80% of *N. lugens* faced mortality during ecdysis, struggling with the shedding of old cuticles and failing to complete the transition into adults [14]. In *B. mori*, the full-length cDNAs (designated as epM) derived from epidermal cells during the last larval molt revealed the presence of 17 RR-2 and all four Tweedle protein transcripts, underscoring the critical role played by these RR-2 and Tweedle (*BmorCPT1*-*BmorCPT4*) genes in silkworm molting [39]. Studies in *A. mellifera* have also shed light on the significance of these gene families during molting stages. Here, five RR-2 genes, two Tweedle genes, and one CPF gene displayed differential expression in the thoracic dorsum during pupal-to-adult molting stages [40]. In *A. gambiae*, *CPF1* and *CPF2* were predominantly expressed towards the end of the fourth instar larvae, whereas *CPF3* and *CPF4* exhibited expression prior to adult emergence [24]. *Apolygus lucorum* is an incomplete metamorphosed insect that undergoes five molting stages from eggs to adults, with minor morphological distinctions between nymphs and adults. Therefore, the prominent expression pattern of CP genes within the RR-2, Tweedle, and CPF families during nymph stages strongly suggests their close involvement in the molting process of *A. lucorum* nymphs.

In addition to the high expression pattern observed during nymph stages, *A. lucorum* exhibits another expression pattern that is prevalent across all developmental stages and tissues, encompassing the CPAP3 subfamily. The CPAP family, ranking as the second largest among CP families and constituting another class of chitin-binding proteins, appears to have a broader taxonomic distribution. Given that chitin is an essential component of the arthropod cuticle, these diverse CPs likely play a role in chitin binding and contribute to the development of arthropods. Numerous CPAP3 proteins are documented to play a fundamental and integral role in upholding the structural integrity of the epidermis across various anatomical regions of insects. For instance, experiments involving RNA interference with *TcCPAP3-A1* in *T. castaneum* resulted in adult lethality [41]. Compared to the control group treated with dsRNA *TcVer*, injecting dsRNA for *TcCPAP3-A1* during the larval stage led to depletion of fat bodies in one-week-old adults, making it impossible to dislodge fecal pellets from their anuses. RNAi for *TcCPAP3-C* resulted in molting arrest from pupal to adult and 100% lethality [20]. In *N. lugens*, *NlugCPAP3-C* (encoding two alternative splicing transcripts, *NlugCPAP3-C5a* and *-C5b*) exhibited a lethal phenotype upon injection with dsRNA during the second-instar nymph stage. Knocking down the common area of *NlugCPAP3-C5a* and *-C5b* or the specific area of *NlugCPAP3-C5b* resulted in very thin body shape and ~90% mortality rate after adult emergence [14]. *CPAP3-E* in *A. gambiae* has been linked to insecticide resistance [41]. This expression pattern of CPAP3 is indicative of a wide array of functions in *A. lucorum*, encompassing aspects such as epidermal integrity, development, and insecticide resistance.

The cuticle is the first line of defense for insecticides to enter the body of insects, and the absorption of insecticides can be reduced by thickening or altering the chemical composition of the cuticle [42,43]. Although cuticular resistance has not yet been adequately characterized at the molecular level, several examples of putative CPs as potential participants in insecticide resistance have been reported. For example, in chlorpyrifos-resistant strains of *P. xylostella*, over 18 CP genes exhibited more than a two-fold upregulation, with some showing increased expression following exposure to chlorpyrifos [6]. In *Culex pipiens pallens,* transcription levels of *CPLCG5* were elevated in both laboratory and field deltamethrin-resistant strains, and knockdown of *CPLCG5* increased susceptibility to insecticides [44]; The expression levels of *CPR47* and *CPR63* are increased in deltamethrin-resistant strains, and knockdown of these two CPR genes by dsRNA injection resulted in increased insect mortality after insecticide treatment [45]; A recent study showed that consumption of *CPLCG5* protein via RNAi reduced cuticle thickness and heightened insecticide sensitivity [46]. Additionally, *CPAP3-E* and *CPLCX1* in *MRS* strains of *A. gambiae* were up-regulated after insecticide exposure, and microscopic analysis revealed that the cuticle in *MRS* strains was significantly thicker than that in susceptible strains [41]. In our study, we observed 75 up-regulated CP genes under different insecticides and temperatures, predominantly belonging to the CPR family. This aligns with previous findings suggesting that CPR, characterized by chitin-binding domains, may enhance the chitin protein matrix in the cuticles to reduce epidermal permeability. As described by An et al. [36], imidacloprid is a positive temperature coefficient insecticide; this aligns with our findings, as more CPs responded to imidacloprid treatment at 35 °C compared to 15 °C. Importantly, under high-temperature conditions, the insect body is subjected to both enhanced insecticide toxicity and heat stress; therefore, these up-regulated genes may represent a combined response to these two pressures. However, at 25 °C, the response pattern deviated from the expected behavior of imidacloprid as a positive temperature coefficient insecticide, which is characterized by increasing toxicity with rising temperature. This discrepancy may be attributed to the proximity of 25 °C to the laboratory rearing temperature of *A. lucorum* (25 ± 1 °C) [36]. At this near-optimal condition, basal metabolism is well balanced, gene expression remains relatively stable, and the detoxification enzyme system functions at peak efficiency. As a result, imidacloprid can be effectively metabolized without extensive transcriptional changes. Consequently, cellular homeostasis is maintained efficiently, and large-scale stress responses, including the induction of CPs, are not activated. In contrast, no CP responses were observed in *A. lucorum* treated with beta cypermethrin, a negative temperature coefficient insecticide, or phoxim, an insecticide without significant temperature-dependent efficacy, at 35 °C. The absence of such a CP response aligns with the mode of action of these two insecticides, reflecting a normal physiological state. Furthermore, A considerable portion of the responsive CP genes belong to the RR-2 subfamily, known for its numerous tandem gene duplicates. Such gene amplification and duplication are often linked to insecticide resistance and may increase the number of transcripts of genes involved in insecticide metabolism. For instance, duplication of the GSTe gene in *Anopheles stephensi* led to an increased gene dose in mosquitoes, possibly an adaptive response to elevate detoxifying enzyme levels in response to insecticide pressure [47]. *Apolygus lucorum’s* host species are highly diverse, primarily phytophagous, and their growth is frequently influenced by plant secondary metabolites, some of which can be detrimental to the insects. Consequently, the expansion of CPs may enhance *A. lucorum’s* metabolic detoxification capabilities. It should be noted, though, that given the limitations of the sample data, the up-regulation of these resistance-associated CPs may be confined to nymphs. A more comprehensive analysis of resistance-related CP genes in *A. lucorum* warrants further investigation across various developmental stages, time points, tissues, and diverse insecticide types.

## 4. Methods and Materials

### 4.1. Identification and Annotation of CP Genes on Genome-Wide Scale

The *A. lucorum* genome has been available from the National Center for Biotechnology Information Search database (NCBI) (https://www.ncbi.nlm.nih.gov/genome/?term=apolygus+lucorum) (accessed on 7 April 2022). Firstly, the potential CPs of *A. lucorum* were identified with previously characterized conserved motifs [16]: the largest CP family, CPR, was recognized based on the extended R&R motif (PF00379) by using HMMER v3.3.2 (http://www.hmmer.org/) (accessed on 25 May 2022) based on Pfam31.0 [48]. The CPR family was further classified into RR-1 and RR-2 subfamilies using the cuticleDB website (http://bioinformatics.biol.uoa.gr/cuticleDB/) (accessed on 17 September 2022) [49]. Tweedle genes were predicted based on the Pfam motif (PF03103). For the CPAP family, the ChtBD2 domain (PF01607) was used to identify putative CPAP genes, then CPAP sequences were further divided into CPAP1 and CPAP3 [29]. The 44 amino acid motif (VSxYSKAVDTPFSSVRKxDxRIVNxA), the C-terminal conserved sequence (LxYSAAPAVSHVAYxGxGxxYGW), the C-X5-C motif (YPAGVNPAACPNYPYCD) [50], and the 18 amino acid motif (PVDTPEVAAAKAAHFAAH) were used to identify the CPF, CPFL, CPCFC, and 18aa family genes, respectively. The remaining CPLCP, CPG and CPH proteins were identified by performing a BLASTp search (E-value ≤ 1 × 10^−5^) against the *A. lucorum* proteome, using validated CP sequences from *B. mori* and *D. melanogaster* as queries (Table S1). Finally, the signal peptides of these putative CPs were detected by SignalP 5.0 (https://services.healthtech.dtu.dk/service.php?SignalP-5.0) (accessed on 3 April 2023) [51].

### 4.2. Phylogenetic Analysis

Phylogenetic analysis was carried out with the corresponding amino sequences from CPR, TWDL, and CPF families. According to relevant literature, the study collected CP sequences from two hemipteran insect species, *N. lugens* [14] and *A. pisum* [27], as well as CPAP protein sequences from multiple insect species [28]. Additionally, since *D. melanogaster* is a well-established model organism in insect research with the most comprehensive genomic and functional annotations, its cuticular protein sequences were also retrieved and downloaded from the FlyBase (https://flybase.org/) (accessed on 14 June 2022) database to serve as a reference in the analysis. Comparison analyses from *A. lucorum*, *D. melanogaster*, *N. lugens*, and *A. pisum* were carried out to analyze the evolutionary placement of CP genes in *A. lucorum*. For the CPR family, the extended R&R consensus was used for phylogenetic analysis, and then, RR-1 and RR-2 subgroups were reconstructed separately. In order to explore the evolution and conservation of CBDs in different insect taxa, phylogenetic trees were reconstructed for CPAP1 and CPAP3 proteins from *A. lucorum* and the other 12 and 9 species, respectively [21]. Sequences were aligned using MAFFT v7 [52], phylogenetic trees were reconstructed using the maximum likelihood (ML) method with the IQ-TREE [53]. The confidence of various phylogenetic lineages was assessed by bootstrap analysis with 1000 replications. For convenience, in terms of their classification and evolutionary relationships, the CPs of *A. lucorum* were renumbered according to their families. For *Drosophila*, the name of CPs was used; for the remaining species, the protein names were the same as previous work. Finally, we used ITOL v6 for editing and coloring the trees (https://itol.embl.de/) (accessed on 23 February 2023).

### 4.3. Expression Profile Analysis of Cuticular Protein Genes

To investigate the CP gene expression profile among the developmental stages of *A. lucorum*, 39 transcriptome data had been prepared from various tissues (leg, head, body, mouthpart, wing, and gut) and developmental stages (eggs, nymphs, and adults) were downloaded from the NCBI SRA database (Accession: PRJNA526332). Each sample contained three biological replicates. We first downloaded the SRA data and then utilized an SRA-Toolkit to divide the paired-end reads. Clean reads were produced from the raw data using Trimmomatic [54] to filter out reads with quality scores lower than 10 and adaptor sequences. To examine gene expression profiles, clean reads of each sample were mapped to *A. lucorum* gene sets using hisat2 [55], and then the TPM value of each putative CP gene was determined using featureCounts [56]. The “weighted trimmed mean of M-values” (TMM) approach from the R package EdgeR [57] was used to normalize the count matrix. Finally, the heatmap was visualized in ITOL (https://itol.embl.de/) (accessed on 23 February 2023) [58] using the normalized matrix. Each sample’s value was calculated using the average of three different biological replicates.

### 4.4. Classification of Gene Duplication Events

The duplication types of CP genes were classified by MCScanX v1.0.0 software (https://github.com/wyp1125/MCScanX) (accessed on 15 March 2023) [59]. Firstly, genome-wide BLASTp analysis was used to determine the homology of different genes in the genome of *A. lucorum* with a cut-off E-value of 1 × 10^−5^. Then, by combining the chromosomal location information of all genes obtained from the genome annotation file with the homology of different genes, all genes can be classified into multiple duplication types, including segmental duplication, tandem duplication, etc. Finally, the duplication types of CPs were extracted based on these results. All visualized works were performed in TBtools (https://github.com/CJ-Chen/TBtools) (accessed on 15 March 2023) [60].

### 4.5. RNA-Seq Analysis of Potential Resistance-Associated CPs

This study used available *A. lucorum* transcriptome data (https://www.ncbi.nlm.nih.gov/sra?term=SRP228853) (accessed on 25 July 2022) from An et al. [36] to identify insecticide resistance-associated CPs. Transcriptome data from this experiment were sequenced after sub-lethal dosage treatments with imidacloprid (IM), beta-cypermethrin (BCP), and phoxim (PHX) at 15 °C, 25 °C, and 35 °C, respectively. These samples were called IM15, IM25, IM35, BCP15, BCP25, BCP35, PHX15, PHX25, and PHX35. The same procedure as in 2.3 was applied to these transcriptome data analysis results to identify differentially expressed genes (DEGs), which were then compared to the discovered *A. lucorum* CP genes. The criteria for screening differentially expressed genes were |log2FC| > 1 and a false discovery rate (FDR/Padj) < 0.05. The heatmap was visualized in TBtools (https://github.com/CJ-Chen/TBtools) (accessed on 8 March 2023).

## 5. Conclusions

Based on genomic data, 211 putative genes encoding CPs were systematically identified in *A. lucorum*. In terms of number, the CPs of *A. lucorum* expanded to some extent. Among them, the occurrence of gene clusters in three families (RR-2, Tweedle, and CPF) is one of the reasons for CP expansion. In particular, the expansion of the RR-2 subfamily may be an evolutionary model for the adaptation of *A. lucorum* to its environment, which can serve as a target for further research on its species differentiation. In *A. lucorum*, two primary expression patterns were identified. One pattern exhibits high expression during the nymph stages, likely closely linked to the molting process in *A. lucorum* nymphs. The other pattern demonstrates sustained high expression throughout all stages, suggesting a broader range of functions. Moreover, when analyzing transcriptome data from samples treated with various pesticides, the elevated expression of certain CPs in specific samples underscores their role in metabolic detoxification. This study offers a comprehensive identification of CP genes within *A. lucorum*, alongside an examination of their expression across various tissues. It lays the groundwork for further research into the evolution and functions of CPs in *A. lucorum*.

## Figures and Tables

**Table 1:** The number of cuticular protein genes of each family in comparison with other insects.

Families	Hemiptera			Diptera					Lepidoptera		Hymenoptera		Coleoptera
	Aluc	Nlug	Apis	Dmel	Asin	Cqui	Aaeg	Agam	Bmor	Msex	Amel	Nvit	Tcas
CPR	144	93	125	101	158	176	244	156	155	205	38	69	110
Tweedle	7	3	3	27	10	9	9	12	4	4	2	2	2
CPAP1	20	17	11	16	0	10	14	13	15	15	14	16	15
CPAP3	7	11	8	7	7	8	9	8	9	10	7	6	7
CPLCA	0	0	0	11	3	3	3	3	2	0	0	0	1
CPLCG	0	0	0	3	26	41	16	27	0	0	0	0	2
CPLCW	0	0	0	0	9	0	7	9	0	0	0	0	0
CPLCP	2	6	0	5	21	0	25	28	7	0	2	4	4
CPCFC	1	0	1	1	0	1	1	1	1	1	0	0	2
CPF	7	2	2	3	4	5	5	4	1	1	3	5	5
CPFL	2	0	0	0	8	0	12	7	4	6	0	0	3
CPG	2	3	0	0	0	0	0	0	23	0	0	0	0
Apidermin	0	0	0	0	0	0	0	0	0	0	3	3	0
Total	211	135	150	175	250	253	345	268	260	246	69	105	151

## Data Availability

All data are contained within the article or its Supplementary Materials as figures or tables.

## Supplementary Materials

The following supporting information can be downloaded at: https://www.mdpi.com/article/10.3390/ijms27073178/s1.
