# Supplementary material for: Identification and Characterization of Cuticular Proteins in the Miridae Insect Apolygus lucorum
Source: Int J Mol Sci. 2026 Mar 31;27(7):3178. doi: 10.3390/ijms27073178 (PMC13073896; doi:10.3390/ijms27073178)
Supplement: Supplementary file 1 [file ijms-27-03178-s001.zip › ijsm-4103113-supplementary/ijms-4103113-supplementary figures with caption-main .pdf]

International Journal of
Molecular Sciences

MDPI

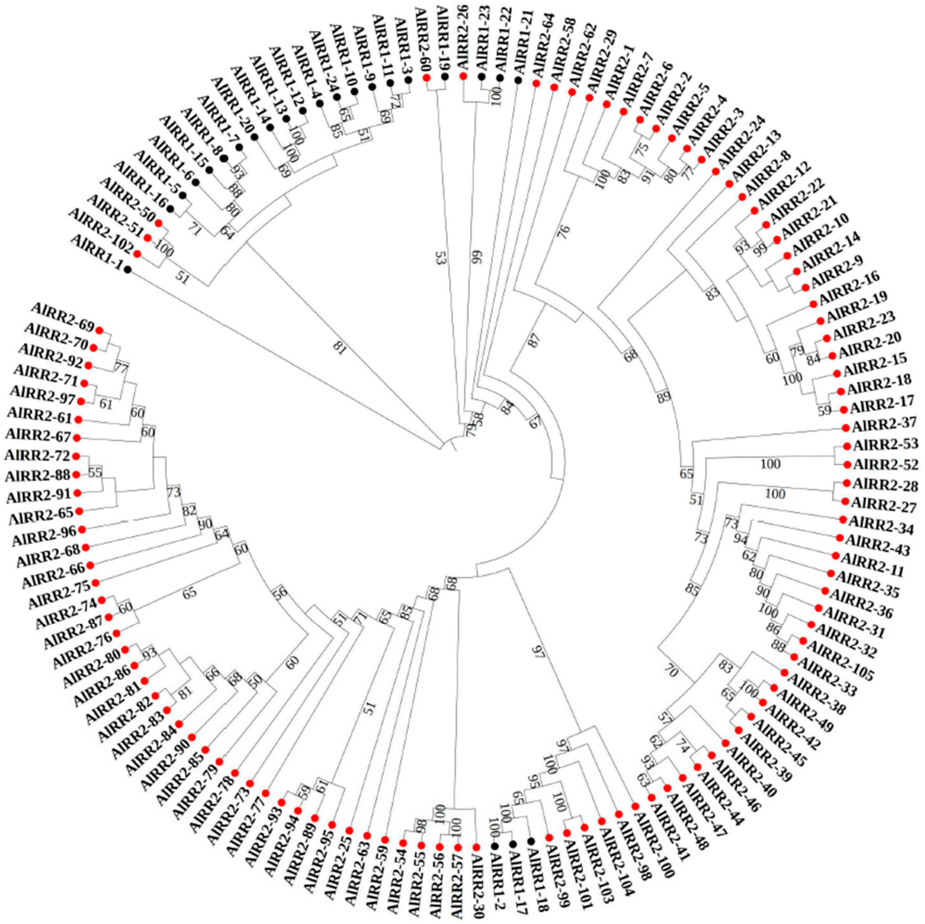

Figure S1 Phylogenetic tree reconstruction of RR-1 (black circle) and RR-2 (red circle) proteins from *A. lucorum* inferred from maximum likelihood (ML). The numbers on the tree were the bootstrap values (below 50 are not shown).

 https://doi.org/10.3390/ijms27073178

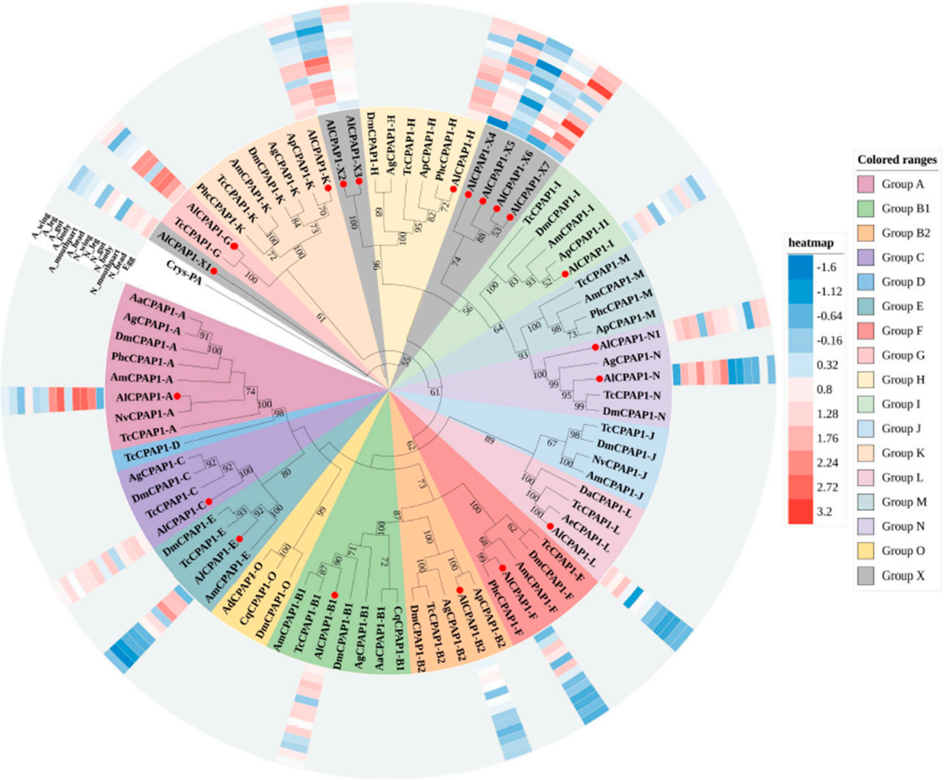

Figure S2 Phylogenetic analysis of CPAP1s from *A. lucorum* and other twelve different insect species: *Acromyrmex echinatior* (Ae), *Acyrthosiphon pisum* (Ap), *Aedes aegypti* (Aa), *Anopheles darlingi* (Ad), *Anopheles gambiae* (Ag), *Apis mellifera* (Am), *Culex quinquefasciatus* (Cq), *Drosophila ananassae* (Da), *Drosophila melanogaster* (Dm), *Nasonia vitripennis* (Nv), *Pediculus humanus corporis* (Phc), *Tribolium castaneum* (Tc). CPAP1 genes from *A. lucorum* are indicated with red circle. The numbers on the tree were the bootstrap values (below 50 are not shown). The tree was rooted by the *D. melanogaster Crys-PA*. The expression profile of *A. lucorum* CPAP1 genes from different tissues is displayed on the right side of the phylogenetic tree. The transcription level of each gene is represented by a square with a color that codes for the values of lg (TPM + 1). Red indicates high expression, whereas blue represents low expression. N, nymph; A, adult.

https://doi.org/10.3390/ijms27073178
