# Supplementary figures and images for: Identification and Characterization of Cuticular Proteins in the Miridae Insect Apolygus lucorum
Source: Int J Mol Sci. 2026 Mar 31;27(7):3178. doi: 10.3390/ijms27073178 (PMC13073896; doi:10.3390/ijms27073178)

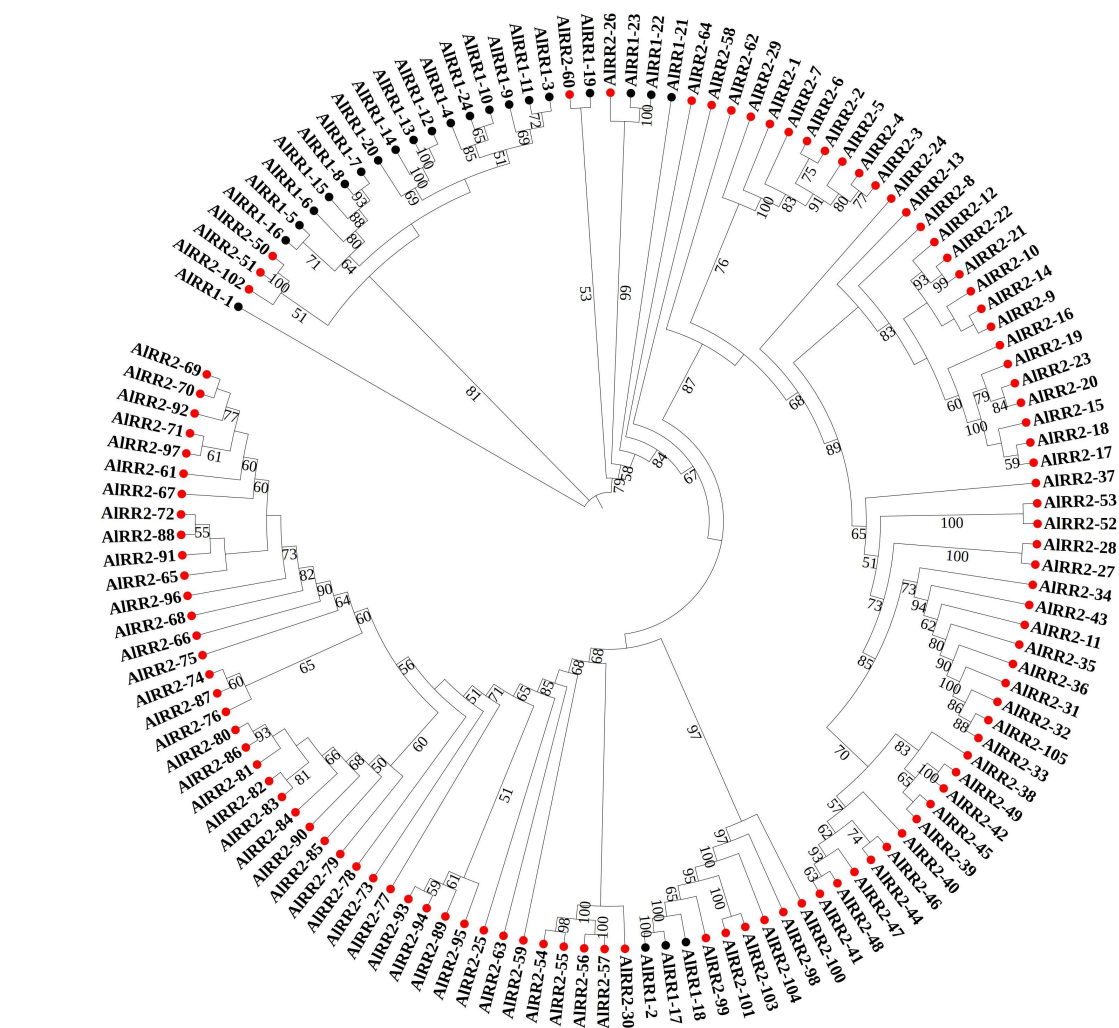

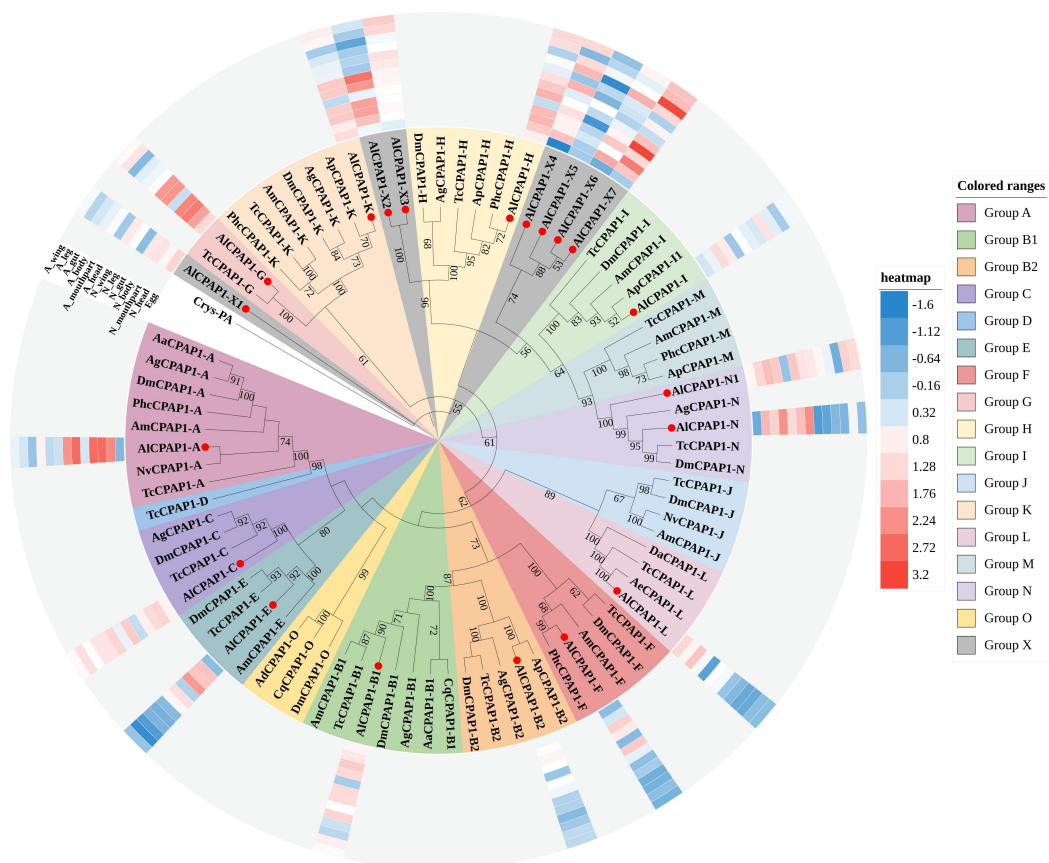

Supplement: Supplementary file 1 [file ijms-27-03178-s001.zip › ijsm-4103113-supplementary/ijms-4103113-supplementary figures.pdf]
